# The Addition of Hydroxyapatite Nanoparticles on Implant Surfaces Modified by Zirconia Blasting and Acid Etching to Enhance Peri-Implant Bone Healing

**DOI:** 10.3390/ijms25137321

**Published:** 2024-07-03

**Authors:** Ricardo Alves Toscano, Stéfany Barbosa, Larissa Gabriele Campos, Cecília Alves de Sousa, Eduardo Dallazen, Carlos Fernando Mourão, Jamil Awad Shibli, Edilson Ervolino, Leonardo P. Faverani, Wirley Goncalves Assunção

**Affiliations:** 1 Department of Diagnosis and Surgery, Sao Paulo State University—UNESP, Aracatuba School of Dentistry, Sao Paulo 16015-050, Brazil; ricardoalvestoscano@gmail.com (R.A.T.); stefany.barbosa@unesp.br (S.B.); larissa.g.campos@unesp.br (L.G.C.); eduardo.dallazen@unesp.br (E.D.); 2 Department of Dental Materials and Prosthodontics, Sao Paulo State University—UNESP, Aracatuba School of Dentistry, Sao Paulo 16015-050, Brazil; cecilia.sousa@unesp.br (C.A.d.S.);; 3 Department of Periodontology, School of Dentistry, Tufts University, Boston, MA 02111, USA; carlos.mourao@tufts.edu; 4 Dental Research Division, Department of Periodontology and Oral Implantology, University of Guarulhos (UnG), Guarulhos 07115-230, Brazil; jshibli@ung.br; 5 Department of Basic Science, Sao Paulo State University—UNESP, Aracatuba School of Dentistry, Sao Paulo 16018-800, Brazil; e.ervolino@unesp.br

**Keywords:** bone regeneration, osseointegration, hydroxyapatite

## Abstract

This study investigated the impact of adding hydroxyapatite nanoparticles to implant surfaces treated with zirconia blasting and acid etching (ZiHa), focusing on structural changes and bone healing parameters in low-density bone sites. The topographical characterization of titanium discs with a ZiHa surface and a commercially modified zirconia-blasted and acid-etched surface (Zi) was performed using scanning electron microscopy, profilometry, and surface-free energy. For the in vivo assessment, 22 female rats were ovariectomized and kept for 90 days, after which one implant from each group was randomly placed in each tibial metaphysis of the animals. Histological and immunohistochemical analyses were performed at 14 and 28 days postoperatively (decalcified lab processing), reverse torque testing was performed at 28 days, and histometry from calcified lab processing was performed at 60 days The group ZiHa promoted changes in surface morphology, forming evenly distributed pores. For bone healing, ZiHa showed a greater reverse torque, newly formed bone area, and bone/implant contact values compared to group Zi (*p* < 0.05; *t*-test). Qualitative histological and immunohistochemical analyses showed higher features of bone maturation for ZiHa on days 14 and 28. This preclinical study demonstrated that adding hydroxyapatite to zirconia-blasted and acid-etched surfaces enhanced peri-implant bone healing in ovariectomized rats. These findings support the potential for improving osseointegration of dental implants, especially in patients with compromised bone metabolism.

## 1. Introduction

In the context of implant dentistry, the use of titanium (Ti) has been widely investigated in the literature, and currently, more than 98% of osseointegrated implants are made of Ti, especially Ti grade IV (ASTM F67) [1]. The incorporation of elements like silica and iron during the foundry, forging, or lamination process increases the mechanical resistance of the material [2].

In addition to mechanical resistance, it is important to consider the biological interactions necessary to guarantee the long-term success of implant-supported rehabilitations. In this sense, it is of great importance to achieve and maintain osseointegration and stable epithelial integration [3,4]. Osseointegration ensures a rigid support for the prosthetic restoration, providing adequate transfer of forces to the adjacent bone during masticatory function. The epithelial integration of the mucoperi-implant tissue consists of the establishment of an epithelial barrier area, which acts to protect the underlying bone tissue and prevent the penetration of microorganisms [5].

Over the past 50 years, the biomedical industry has continuously modified implants to achieve greater stability, adequate mucointegration, and increased longevity of rehabilitation treatments. Surface texturing processes of dental implants aim to enhance mechanical resistance to corrosion and improve osseointegration by increasing the contact surface area [6,7].Various topographical and physicochemical surface modifications have been developed [8,9,10], including addition techniques like titanium plasma spray (TPS) and hydroxyapatite (HA) coatings [7,11] and subtraction techniques such as electropolishing, mechanical polishing, acid treatments, sandblasting, oxidation, and laser irradiation [12,13,14]. These microtopographic modifications have consistently shown promising results in enhancing osseointegration parameters, including increased removal torque values and improved bone quantity and quality [14].

Studies have shown that roughening the implant surface can enhance the peri-implant microenvironment, leading to improved bone healing [10,14]. The factors that promote better and faster osteogenesis for these surfaces are related to higher blood clot retention, increased migration and proliferation, and increased implant surface contact area [8,9]. The incorporation of elements that precipitate in the bone tissue matrix, such as calcium, phosphorus, or hydroxyapatite, is a very interesting technique, as is the texturing process, which has demonstrated greater affinity with cells fundamental to bone formation [14,15,16]. Among these elements, hydroxyapatite has demonstrated chemical similarity to the inorganic components of bone matrix [17]. It offers advantages such as biocompatibility, slow degradability, and osteoconductive and osteoinductive properties [18]. These similarities and benefits have spurred scientific interest in using hydroxyapatite in bioengineered materials to improve bone tissue healing. Synthesizing hydroxyapatite at the nanoscale is particularly important, as it can be used to functionalize surfaces, such as titanium dental implants, and target specific biological systems, leading to better bone healing [17,19].

For osseointegration to occur, the quality of bone tissue is crucial, as the implant/peri-implant bone complex is subjected to constant thermal, chemical, and mechanical stresses, necessitating continuous remodeling [3,4]. This dynamic process involves coordinated bone resorption, formation, and mineralization. Any imbalance in this process can compromise skeletal tissue quality and lead to pathological conditions [20]. Osteoporosis, characterized by progressive bone loss and deteriorated bone microarchitecture, is a key factor that can impair the success of osseointegrated implants [21,22]. This condition, prevalent in postmenopausal women due to decreased estrogen levels, significantly reduces implant survival rates, despite not contraindicating rehabilitation treatment [23,24,25].

Therefore, it is necessary to carry out studies that seek to optimize the bone healing process around osseointegrated implants. An appropriate experimental animal model for this purpose is the installation of implants in the tibial metaphysis of rats. These rats’ long bones have a microarchitecture similar to human alveolar bone, thus simulating the tissue interactions to be analyzed [26,27,28]. Linked to the installation of implants, bone with a lower density, characterized by experimental osteoporosis, is an interesting model for verifying the influence of implant coating methods, as well as the biological behavior of osseointegration.

As such, this study aimed to analyze the impact of adding hydroxyapatite nanoparticles to implant surfaces previously treated with zirconia blasting and acid etching. Specifically, it investigated the structural changes to the implant and the resulting effects on bone healing parameters in low-density bone sites.

## 2. Results

### 2.1. Structural Characterization

#### 2.1.1. Scanning Electron Microscopy (SEM)

**Figure 1:**
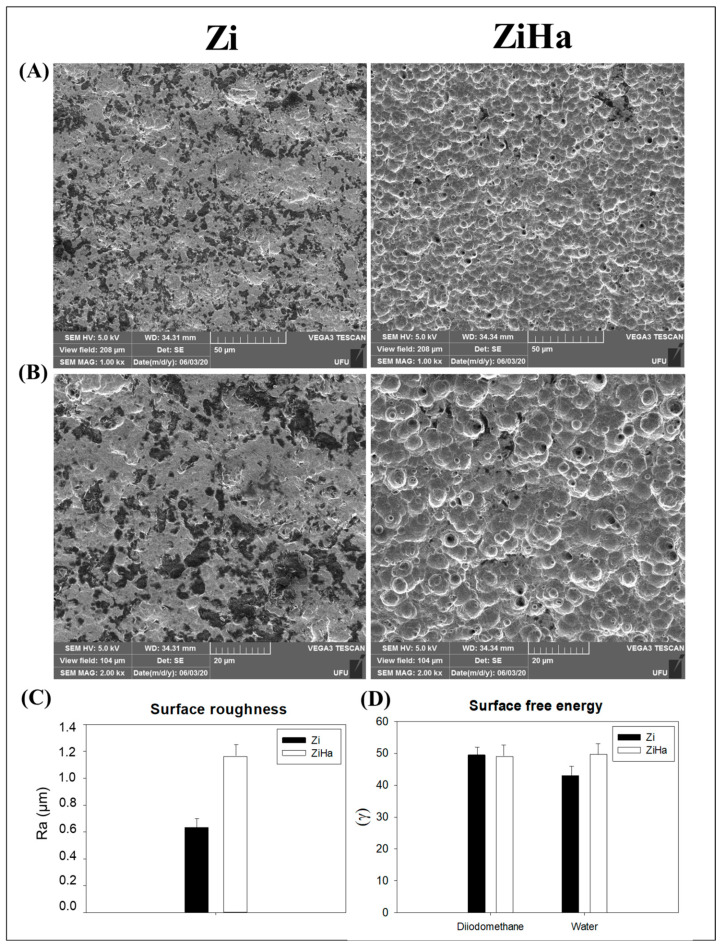
Topographical characterization of Zi and ZiHa surfaces. (**A**,**B**) Photomicrographs related to the analysis of scanning electron microscopy (SEM) (magnification of 1000× and 2000×) of the surface of Ticp discs treated with Zi and ZiHa. (**C**) Graphical representation of the mean surface roughness (Ra) of the Ticp alloy as a function of surface modifications in both experimental groups. (**D**) Graphical representation of the mean surface free energy concerning the contact angle for water and diiodomethane of the Ticp alloy, as a function of surface modifications for the Zi and ZiHa groups) (N = 10 discs per group).

The photomicrographs (SEM, JEOL JSM-6010LA, Peabody, MA, USA) of the Zi group showed an irregular surface, with groove formation indicated by the darker regions. In contrast, the ZiHa group exhibited elevated surfaces with the appearance of congruences and an absence of the grooves found in the other group (Figure 1A,B).

#### 2.1.2. Profilometry—Surface Roughness Analysis

No statistically significant differences were observed in the roughness of the evaluated surfaces. However, there was a tendency for the ZiHa group to exhibit greater roughness than the Zi group (Figure 1C).

#### 2.1.3. Surface Free Energy

The surface free energy analysis evaluated the polar (water) and dispersive (diiodomethane) components of the Ticp alloy and their modifications in relation to the contact angle, revealing similar results for both experimental groups regarding both components (polar and dispersive) (Figure 1D).

### 2.2. Histological Analysis

**Figure 2:**
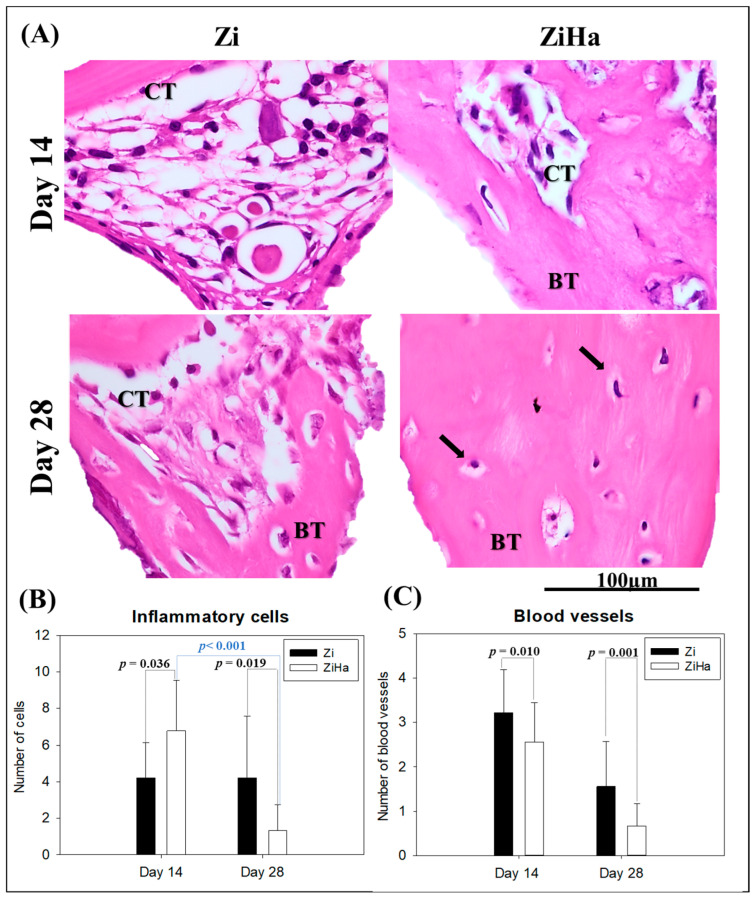
Visual and graphical representation of the histological analysis. (**A**) Photomicrographs for the representation of bone tissue maturation of each experimental group at 14 and 28 days post operation. The black arrows represent the osteocytes in the bone matrix. (**B**) Graphical representation of inflammatory cell count, showing statistical differences between the Zi and ZiHa groups at 14 (*p* = 0.036) and 28 days (*p* = 0.019), as well as for the ZiHa group in both periods (*p* < 0.001). (**C**) Graphical blood vessel count representation, demonstrating an intergroup difference at 14 (*p* = 0.010) and 28 days (*p* = 0.001). (n = 6 tibiae per group). Original magnification: 1000×. BT: Bone tissue; CT: connective tissue.

Through histological analysis of photomicrographs at 1000× magnification (Figure 2A), the characteristics and degree of maturation of the bone tissue whorls formed during implant osseointegration in both groups were observed. At 14 days, it was noted that the Zi group exhibits a greater amount of mature connective tissue with little newly formed bone tissue. In contrast, ZiHa shows a higher amount of newly formed bone interspersed with a small amount of connective tissue. By day 28, Zi demonstrates increased newly formed bone with connective tissue present at the center of bone formation. Meanwhile, ZiHa displays significant bone tissue formation with osteocytes present in the bone matrix and without interspersed connective tissue.

Additionally, histological analysis verified the inflammatory profile in both groups using 1000× original magnification, by which inflammatory cells (Figure 2B) and blood vessels (Figure 2C) were quantified. At 14 days postoperative, a higher quantity of inflammatory cells was observed in the ZiHa group (6.78 ± 2.77) compared to Zi (4.22 ± 1.92) (*p* = 0.036). Meanwhile, at 28 days, a prevalence of inflammatory cells was noted in the Zi group (4.22 ± 3.35) compared to ZiHa (1.33 ± 1.41) (*p* = 0.019). Besides the statistical intergroup difference, there was a significant difference between both measurement points in the ZiHa group (*p* < 0.001). The number of blood vessels was higher in the Zi group at both postoperative time points (day 14—Zi: 3.22 ± 0.97//ZiHa: 2.56 ± 0.88) (*p* = 0.010); (day 28—Zi: 1.56 ± 1.01//ZiHa: 0.67 ± 0.50) (*p* = 0.001).

### 2.3. Immunohistochemistry

**Figure 3:**
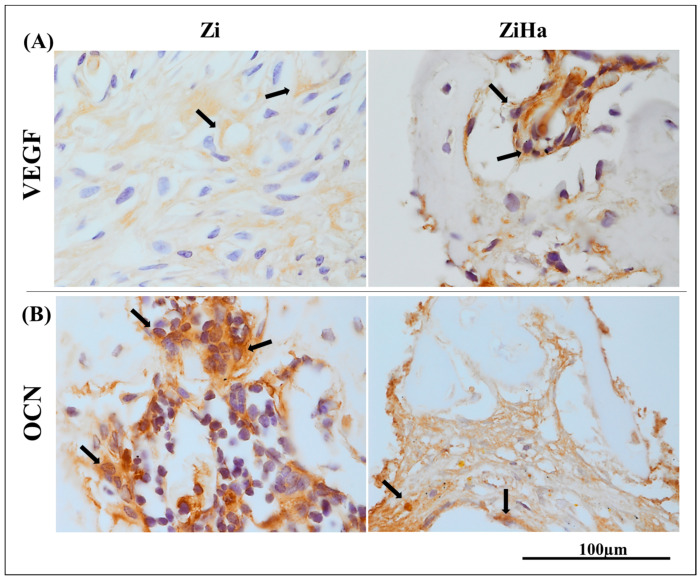
Representative images of immunohistochemical analysis. (**A**) Photomicrographs representing immunostaining for vascular endothelial growth factor (VEGF) at the 14-day time point. (**B**) Photomicrographs representing immunostaining for osteocalcin (OCN) at the 28-day time point. The black arrows represent the cells labeled by the primary antibodies in all images (regions of brown staining). Original magnification: 1000×.

The immunostaining appeared as a brownish color, localized within the cytoskeleton of the cell cytosol for both vascular endothelial growth factor (VEGF) and osteocalcin (OCN), as well as in the extracellular matrix. Figure 3A illustrates the immunostaining patterns at 14 days for VEGF, and Figure 3B at 28 days for OCN

VEGF marked mainly fibroblasts, showing a greater distribution on most slides in the Zi group. Osteoblasts stained with OCN exhibited similar moderate intensity staining in both groups, with more than 50% of the slides showing staining.

### 2.4. Biomechanical Analysis (Reverse Torque)

**Figure 4:**
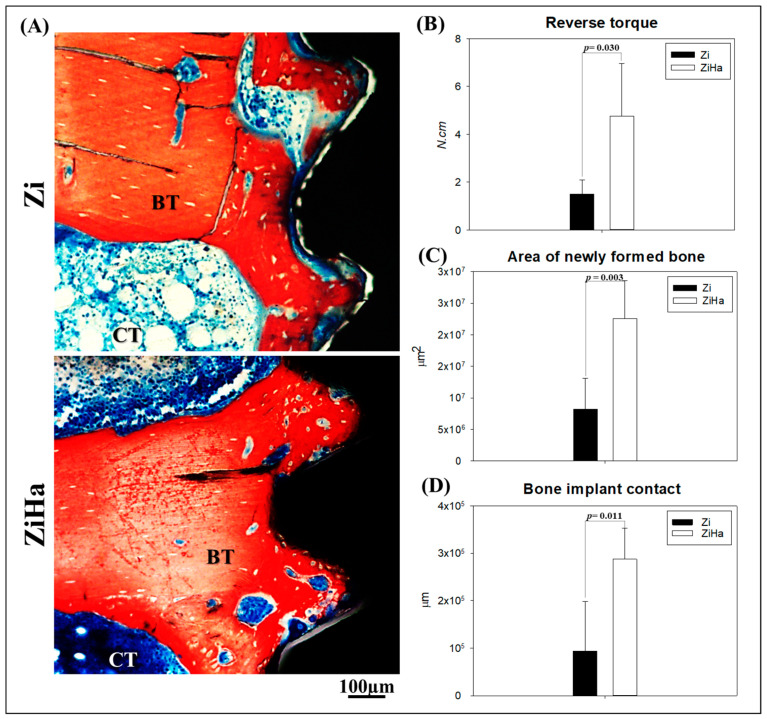
Visual and graphical representation of histometric analysis. (**A**) Representative images of peri-implant bone tissue on calcified slides, 12× objective. (**B**) Graphical representation of the reverse torque analysis, showing superiority in the ZiHa group (*p* = 0.030). (**C**) Demonstration of neoformed bone area values, highlighting greater neoformation in the ZiHa group (*p* = 0.003). (**D**) Representation of bone-implant contact, registering higher values in group ZiHa (*p* = 0.011) (n = 6 tibiae per group).

The biomechanical analysis of counter-torque showed that implants subjected to hydroxyapatite nanoparticles functionalization (ZiHa group) exhibited a higher torque peak (4.75 ± 2.22 N·cm) during the breaking of the bone/implant interface compared to the Zi group (1.5 ± 0.58 N·cm). The intergroup difference was statistically significant (*p* = 0.03) (Figure 4B).

### 2.5. Histometry

The reparative pattern was more favorable in the ZiHa group (Figure 4A). The area of neoformed bone was significantly higher in group ZiHa (2.2 × 10^7^ ± 5.9 × 10^6^ µm^2^) than in group Zi (8.2 × 10^6^ ± 4.8 × 10^6^ µm^2^) (*p* = 0.003) (Figure 4C). Similarly, the bone/implant contact had a significantly higher value in group ZiHa (2.8 × 10^5^ ± 6.5 × 10^1^ µm) compared to the Zi group (9.3 × 10^4^ ± 1.0 × 10^5^ µm) (*p* = 0.011) (Figure 4D).

## 3. Discussion

The results of this study have clarified the optimization properties of peri-implant bone healing related to the surface coating method achieved through zirconia blasting and acid etching with the addition of hydroxyapatite nanoparticles (ZiHa).

It is known that the coating techniques of dental implants directly influence the cell differentiation and calcification of the bone matrix during the phenomenon of osseointegration [9]. In this study, it was observed that the ZiHa coating was able to modify the structural characteristics of the surface, resulting in a more complex morphology with numerous pores resembling “volcanoes,” evenly distributed throughout the sample. The increase in surface roughness, combined with the incorporation of components similar to those naturally present in the organism, such as hydroxyapatite, creates a more favorable environment for blood interaction and, consequently, induction of osteogenic lineage of proteins and cells [14,29,30]. This enhances the likelihood of successful osseointegration [4]. Regarding surface characteristics, its physical properties should be considered. These were very similar to implants currently on the market (Zi) in terms of surface roughness and free energy, reinforcing the feasibility of applying the treatment tested here in implant-supported rehabilitation.

In this context, it is important to remember that for the sake of durability, the surfaces of osseointegrated implants must integrate with three different types of tissue during the initial phases of healing: epithelial, connective, and osseous tissue [31]. Therefore, the inflammatory profile associated with peri-implant healing may predict the treatment-related prognosis [32]. In this study, a quantitative pattern of inflammatory cells was observed, consistent with the healing times in the respective groups. It is expected that in the ZiHa group, the addition of hydroxyapatite to the surface would generate greater inflammation in the early healing periods compared to the Zi group. Considering the improved bone healing observed in the ZiHa group at 14 and 28 days, we do not view the higher number of inflammatory cells as detrimental. Instead, we infer that this inflammatory response likely facilitated the biological pathways and bone cell activity, leading to better osseointegration. At 28 days, a significant reduction in the number of cells could already be observed. Regarding the analysis of blood vessels, the results demonstrated an exponential decrease in the number of vessels as the replacement of connective tissue by neoformed bone occurred, showing a higher number of vessels in the Zi group in both periods.

Regarding the initial integration of the implant with bone tissue, the employed surfaces optimize a series of coordinated events necessary for peri-implant bone neoformation [4,20]. In this sense, the histological analysis of photomicrographs at 1000× magnification revealed that after 14 days, the Zi group still presented quite immature bone tissue, with a large number of cells and connective tissue interspersed in the precipitation regions of the bone matrix. The ZiHa group, on the other hand, showed greater filling by bone tissue, with much more advanced maturation. The same pattern was visible after 28 days, with the ZiHa group presenting bone tissue of a more mature and organized structure.

Understanding the chronological formation of bone tissue provides insight into the criteria used to identify tissue maturation across different groups. Immature bone tissue is characterized by a higher amount of connective tissue that has not yet been replaced by an osteoid matrix and a greater presence of active osteoblasts secreting this matrix. Its architecture is marked by finer and more widely spaced trabeculae. In contrast, mature bone tissue has already secreted an osteoid matrix, with osteoblasts now transformed into osteocytes residing within it, representing the highest degree of cellular differentiation in the osteogenic lineage [33,34,35]. Therefore, mature bone tissue is distinguished by a reduced amount of connective tissue and active osteoblasts and a higher concentration of osteocytes, as observed in the ZiHa group at 28 days. Its architecture includes more trabeculae with closer spacing and a higher mineralization density in the tissue.

The immunohistochemical analysis effectively identified the proposed markers. Immunostaining for VEGF at 14 days was slightly more intense in the Zi group, which demonstrates that the ZiHa group was already in a more advanced phase of bone healing, due to reduced protein expression and consequent reduction of blood vessels in the healing region. With regard to OCN at 28 days post-surgery, it can be affirmed that implant osseointegration in the tibia of osteoporotic rats had occurred in both groups.

Another factor of great importance for the prognosis and longevity of implants concerns primary and secondary stability, which are linked to the quality and quantity of available bone [36,37]. Based on the results obtained in this research, it can be considered that the surface textured by zirconia blasting with acid etching and the addition of hydroxyapatite nanoparticles presents a more favorable prognosis regarding secondary stability. The biomechanical analysis demonstrated that the implants with this surface had a higher torque peak value during rupture of the bone-implant interface. Favorable results regarding secondary stability associated with this surface treatment have also been noted by other authors, thus reinforcing the results of this study [38,39].

Furthermore, as a primary outcome analysis, histometry was crucial in demonstrating favorable bone neoformation in the ZiHa group. A statistically significant intergroup difference was noted in the parameters of neoformed bone area and the extent of bone-implant contact. The addition of hydroxyapatite clearly accelerated bone healing in ovariectomized rats, leading to a favorable bone condition for implant installation, as demonstrated by biomechanical analysis. The same was observed in relation to the linear extent of bone-implant contact. These are encouraging findings that corroborate the existing literature about the reparative response to zirconia blasting and double acid etching [38,39,40].

This study used ovariectomized rats as an animal model to simulate compromised bone metabolism. However, as with all animal model experiments, the results may not directly translate to human patients due to species-specific differences in bone healing and metabolism. While the study provides valuable preclinical evidence, clinical trials are necessary to confirm the effectiveness and safety of the ZiHa surface modification in human dental implant procedures. Untreated Ti surfaces were not used as a control group for most studies, owing to the excellent biocompatibility and enhanced osseointegration noticed with nanohydroxyapatite-functionalized Ti implants [41], and the functionalization tested here also included zirconia blasting. Even so, a limitation to be considered is the lack of a direct comparison between our functionalization method and untreated Ti surfaces (machined surface), especially regarding topographical assessments to show the maintenance of incorporated particles.

The favorable results of this study contribute to the search for alternative means to optimize bone healing, particularly in critical situations like systemic conditions with a poor metabolic bone response. Texturizing through zirconia blasting with acid etching and the addition of hydroxyapatite nanoparticles is an easy and promising treatment, capable of changing the morphology and chemical composition of implant surfaces, as well as accelerating bone tissue maturation. Therefore, it positively influences the secondary stability of osseointegrated implants.

## 4. Materials and Methods

For the present research, the animals were divided into the following two groups: ZiHa, comprised of discs textured using a zirconia blasting method and acid etching with the addition of hydroxyapatite nanoparticles, and the Zi group, including discs textured with zirconia blasting and acid etching. All discs were treated in accordance with the manufacturer’s standards, which constitute the baseline.

### 4.1. Implants/Discs Coating by Zirconia Blasting and Acid Etching with the Addition of Hydroxyapatite Nanoparticles

A total of 20 discs (10 mm in diameter) and 22 Ticp grade 4 microimplants (99.7 Ti; 0.16 O_2_; 0.004 N_2_; 0.006 C; 0.0019 H_2_ and 0.12 Fe in % of weight) 2 mm in diameter and 4.5 mm in length [27], supplied by the company DSP Biomedical (Campo Largo, Brazil), were used. The discs were polished, cleaned in an ultrasonic tank, and dried using hot air jets at 250 °C [42,43]. Next, the discs and implants were randomly divided into two groups, with 10 discs and 11 implants designated for the coating process with zirconia blasting and acid etching (Zi group), while the remaining 10 discs and 11 implants were included in the ZiHa group and subjected to the surface texturing method of zirconia blasting and acid etching, with added hydroxyapatite nanoparticles.

The coating method was initiated by blasting zirconia particles, producing a surface microtexture through subtraction. To this end, the surface was blasted with 70 µm zirconia particles at a distance of 5 cm from the blasting gun nozzle for 20 s at a pressure of 6 Bar, creating surfaces with irregular grooves through abrasion. Blasting was followed by an acid attack on the surfaces, characterizing the acid subtraction process. The elements were placed in a test tube containing a solution of hydrofluoric acid (HF) and nitric acid (HNO_3_) with a concentration of 5/100 for HF and 20/100 for HNO_3_, heated to 60 °C, and left to react for 40 min. The contents of the container were homogenized with a glass rod every 5 min.

After 40 min, the acid was replaced, and the process was repeated under the same parameters, resulting in a total immersion time of 80 min in the acid solution. Following these procedures, the acid was removed, and the implants were immersed in distilled water four times. Subsequently, the implants were placed in a Becker test tube filled with distilled water and transferred to an ultrasonic tank (Setecsom—Mairiporã, São Paulo, Brazil) filled with distilled water, which was changed every 10 min over a duration of 40 min. After the discs and implants were removed from the ultrasonic tank, they underwent steam sterilization in a C 5 boiler (CR—Curitiba, Paraná, Brazil). Finally, the implants were dried in an oven (Odontobrás—Ribeirão Preto, São Paulo, Brazil) for 5 h at 100 °C.

To incorporate hydroxyapatite nanoparticles onto the discs and implants of the ZiHa group, biomimetic processing was conducted [44]. The discs and implants underwent an alkaline phase using an aqueous solution of 1 M NaOH in a vertical autoclave (Fanem—São Paulo, São Paulo, Brazil) at 130 °C for 60 min, followed by drying at room temperature. Thermal treatment of the discs and implants was performed in a tubular oven (EDG—São Carlos, São Paulo, Brazil) at 200 °C for one hour, with a heating rate of 10 °C/min. Subsequently, the discs and implants were immersed in 5 mL of modified simulated body fluid (mSBF) (Jalota et al., 2006) at 37 °C, with the solution changed every two days, and removed after 14 days. Upon removal from the solution, the implants were stored in a desiccator to prevent contamination and washed with distilled water [44].

**Figure 5:**
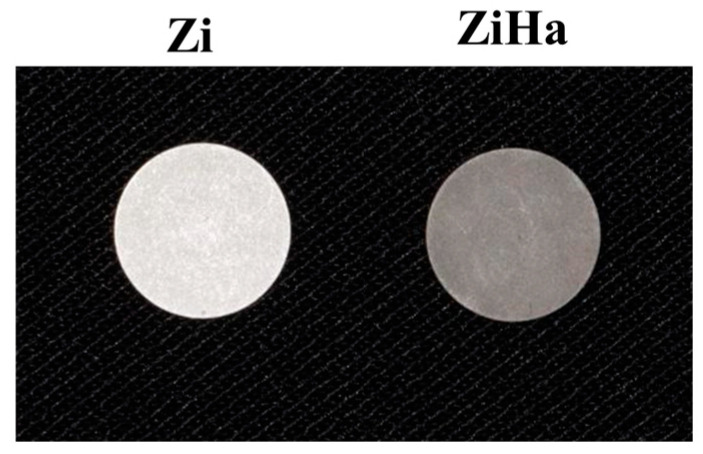
Representative image of the two discs used for surface characterization analysis with their surfaces corresponding to the respective implants.

The preparation of the mSBF solution began with washing all materials using distilled water. Subsequently, the materials were washed with 1M HCl solution and rinsed five times with pure deionized water. Following this, the materials were dried in an oven. Reagents were dissolved in a wide-mouth container with moderate agitation. One-third of the deionized water preheated to 37 °C was added to the flask for dissolution. The solution was stirred for 10 to 15 min to achieve homogeneity. pH adjustment was conducted by transferring the solution at 37 °C into a beaker under moderate agitation and adding preheated water until the volume reached three-quarters of the final volume. The pH was maintained between 7.5 and 8.0, controlled by adding calcium sulfate (Sigma-Aldrich Products, Burlington, MA, USA). The final volume measurement was performed after transferring the solution into a volumetric flask. Deionized water was added to complete the final volume to one liter. After measurement, the flask was capped, and the solution was homogenized by shaking it forty times. Finally, the solution was stored in a cleaned polyethylene bottle in a refrigerator. The representative image of the discs after coatings’methods can be seen in the Figure 5.

### 4.2. Structural Characterization

#### 4.2.1. Scanning Electron Microscopy (SEM)

Scanning electron microscopy (SEM) at magnifications of 1000× and 2000× was used for qualitative characterization of the surface topography. The SEM analysis was performed using a JEOL JSM-6010LA instrument (Peabody, MA, USA) operating at 5.0 kV with a working distance (WD) of 34 mm.

#### 4.2.2. Profilometry—Analysis of Surface Roughness

The surface roughness (Ra—arithmetic mean) was measured using a profilometer (Dektak D-150, Veeco, Plainview, NY, USA) with a cut-off of 500 μm and a constant measuring time of 12 s. Three measurements were obtained for each Ticp disc, and the mean value was calculated [26,45].

#### 4.2.3. Surface Free Energy

Surface free energy was measured following the protocol suggested by Combe et al. (2004) [46], using a goniometer (Ramé-Hart Instrument Co., Succasunna, NJ, USA). To determine the surface free energy, the contact angle formed between the disc surface and a sessile drop of test liquids, including diiodomethane (Sigma-Aldrich Products, Burlington, MA, USA) and distilled deionized water, was measured. This measurement involved applying three drops of each liquid in an environment with a temperature of 21 ± 1 °C and controlled humidity [46].

### 4.3. In Vivo Study: Experimental Design

To evaluate in vivo peri-implant bone healing, bilateral ovariectomy was performed on all rats to induce osteoporosis. After 90 days, the animals underwent surgery for implant installation in the tibias of both ZiHa and Zi groups, chosen at random.

The study was conducted in compliance with the Ethical Principles for Animal Experimentation and approved by the Ethics Committee on Animal Use at the School of Dentistry, Araçatuba, Brazil (São Paulo State University—UNESP, case no. 00528-2018).

Twenty-two adult female Wistar rats (*Rattus norvegicus albinus*) (n = 22 tibias per experimental group), aged six months, with body weights ranging from 250 to 300 g, were used. Throughout the experiment, the animals were housed in cages under controlled conditions with a stable temperature (22 ± 2 °C) and a controlled light cycle (12 h light and 12 h dark). They were fed with solid feed (Ração Ativada Produtor^®^, Anderson & Clayton S.A.—Laboratório Abbot do Brasil Ltd.a, São Paulo, Brazil) throughout the experiment and had access to water ad libitum, except for a 12 h period before surgical procedures.

For sample size calculation, the “Sample Size for ANOVA” tool in SigmaPlot 12.0 (Systat Software Inc., San Jose, CA, USA) was used, with Polo et al. (2020) serving as a reference due to their similar methodology [29]. Based on the results from a primary outcome analysis (histometric analysis) showing an intergroup mean difference of 144.3, a standard deviation of 41.13, and a statistical power of 95%, a minimum sample size of 4 tibias per group was determined. Accordingly, four animals (n = 4 tibias per group) were allocated for biomechanical analysis, and six animals (n = 6 tibiae per group) were designated for other procedures, including analysis of calcified tissue (histology and immunohistochemistry) at 14 and 28 days, and histometry at 60 days, totaling 22 animals.

### 4.4. Ovariectomy Procedure

All rats underwent bilateral ovariectomy under sedation, following established protocols [27,47]. After 90 days, osteopenia was induced [29,47].

### 4.5. Implant Surgery Sequence

Ninety days after ovariectomy surgery, all animals fasted for 12 h prior to the implantation procedure in the tibiae [29]. The surgical procedures for implant installation followed established protocols [29,47,48].

Microimplants with both surfaces Zi and ZiHa were placed in each tibial metaphysis. The implants had dimensions of 2 mm in diameter and 4.5 mm in height and were sterilized by gamma rays. The choice of tibia was randomized through the website www.randomization.com (accessed on 27 June 2024). The muscle and superficial tissues were sutured using resorbable (Poliglactin 910; Vycril—Johnson & Johnson, São José dos Campos/SP, Brazil) and non-resorbable (Nylon 4-0—Johnson & Johnson, São José dos Campos/SP, Brazil) sutures.

### 4.6. Peri-Implant Bone Healing Assessment

At 14 and 28 days postoperatively, a portion of the animals were euthanized using an anesthetic overdose (Sodium Thiopental, 100 mg/kg), administered intramuscularly. The collected samples were then processed for analysis in decalcified tissues (histological and immunohistochemistry). After 28 days, four animals were euthanized for reverse torque analysis, while at 60 days postoperatively, the remaining animals were euthanized and the samples were submitted to histometric analysis.

#### 4.6.1. Analyses for Decalcified Tissues

##### Histological Analysis

On days 14 and 28, six samples from each group were obtained and decalcified for 90 days. Subsequently, the pieces were dehydrated, cleared in xylene, and embedded in paraffin, resulting in the production of slides with 5 μm thick sections. After microtomy, the paired slides were stained with hematoxylin and eosin (HE), and the chronology of bone healing in the peri-implant region was analyzed. The HE-stained slides were photomicrographed at the region of the most central gyri to observe the maturation and characteristics of the bone tissue in the different groups. Photomicrographs were captured at 1000× magnification using a digital camera (JVCKENWOOD USA Corporation—JVC TK-1270 Color Video Camera, Long Beach, CA, USA) attached to an optical microscope (Axiolab, Zeiss, Oberkochen, Germany) connected to a computer.

To evaluate the inflammatory profile, with emphasis on lymphocyte count and the number of blood vessels, three photomicrographs were taken at 1000× magnification of each sample from different regions of the peri-implant area. Next, the images were analyzed using the “Grid” tool of ImageJ software (ImageJ 1.52a—National Institutes of Health, Bethesda, MD, USA). A total of 130 intersection points were used to facilitate the visualization of cells and blood vessels. Additionally, the “Cell Counter” tool was used to count the mentioned structures.

##### Immunohistochemical Reactions

Of the prepared slides that were decalcified and embedded in paraffin, the odd-numbered ones were assessed for immunohistochemical reactions, while endogenous peroxidase activity was inhibited using hydrogen peroxide. Subsequently, the slides underwent an antigen retrieval step with citrate phosphate buffer (pH 6.0). The primary antibodies used were anti-vascular endothelial growth factor (anti-VEGF) (SC-7269, Santa Cruz Biotechnology^®^, Dallas, TX, USA) at 14 days postoperatively and osteocalcin (OCN) (SC-18319, Santa Cruz Biotechnology^®^, Dallas, TX, USA) at 28 days.

The secondary antibody used was biotinylated anti-goat produced in rabbits (Pierce Biotechnology, Waltham, MA, USA), streptavidin and biotin amplifier (Agilent Dako Pathology Lab Solutions, Santa Clara, CA, USA), and diaminobenzidine (Agilent Dako Pathology Lab Solutions) as the chromogen. For each antibody, protein expression was evaluated semi-quantitatively (qualitative ordinal analysis) by assigning different scores based on the number of immunostained cells in the healing process. The semi-quantitative analysis was conducted using an optical microscope (Leica DMLB, Heerbrugg, Switzerland) [49,50].

#### 4.6.2. Analyses for Calcified Tissues

##### Biomechanical Analysis of Implants (Reverse Torque)

For the biomechanical analysis, after euthanizing the animals at 28 days post operation, all tibial metaphysis were accessed to expose the implants and conduct reverse torque testing. An implant mount was adapted to the hexagon of the implants, and a digital torque meter was attached to the implant mount (Portable Digital Torque Wrench Model TQ-680, Instrutherm Measurement Instruments, São Paulo, Brazil).

A counterclockwise movement was applied, increasing the reverse torque until the implant rotated within the bone tissue, completely breaking the bone-implant interface. At this point, the torque meter recorded the maximum torque peak for this rupture in Newton-centimeters (N-cm).

##### Histometric Analysis

For the histometric analysis, six animals were euthanized 60 days post-surgery. After fixation, the specimens were dehydrated using an increasing alcohol sequence, with the solution changed every three days. The dehydrated specimens were placed in an orbital shaker (KLine CT-150, Cientec—Laboratory Equipment, Piracicaba, SP, Brazil) daily for 4 h. Upon completion of dehydration, the specimens were immersed in a mixture of 100% alcohol and Technovit photopolymerizable resin (Heraeus Kulzer GmbH, Wasserburg am Inn Germany) at varying concentrations until only the resin remained as the immersion medium. Subsequently, the specimens were embedded in Technovit resin, cured under light, and processed according to the Exakt protocol (Cutting System, Apparatebau GmbH, Hamburg, Germany). They were then sectioned and ground using an automatic cutting and polishing system until sections approximately 80 μm thick were obtained.

Following this, the slides were stained with alizarin red and Stevenel’s blue. Bone-implant contact (BIC) and the area of newly formed bone (NFB) around the most central implant thread on each side were calculated. The slides were photomicrographed and saved as TIFF files, which were then opened in ImageJ software (ImageJ 1.52a—National Institutes of Health, Bethesd, USA). BIC was measured in micrometers using the “straight” tool, while the “freehand” tool was utilized to measure the NFB area in square micrometers (μm^2^).

### 4.7. Statistical Analysis

For each quantitative parameter in the results, the intergroup difference (ZiHa vs. Zi) was evaluated using SigmaPlot 12.3 (Systat Software Inc., San Jose, CA, USA). For topographical analysis (mean roughness and surface free energy), area of newly formed bone, bone-implant contact, and torque resistance, Student’s *t*-test was applied. Two-way ANOVA followed by Tukey’s post-test was employed for other parameters at a significance level of *p* < 0.05.

## 5. Conclusions

In conclusion, adding hydroxyapatite nanoparticles to zirconia-blasted and acid-etched surfaces enhances peri-implant bone healing in ovariectomized rats. The ZiHa group showed significant improvements in surface morphology, reverse torque, newly formed bone area, and bone/implant contact values compared to the Zi group. Histological and immunohistochemical analyses indicated higher bone maturation for ZiHa at 14 and 28 days. This coating method is promising for optimizing bone healing, especially in low bone quality sites and conditions with poor metabolic bone response.

## Data Availability

The authors confirm that the data supporting the findings of this study are stored in an online repository and are available upon request.
